# Neutrophil extracellular traps in influenza infection

**DOI:** 10.1016/j.heliyon.2023.e23306

**Published:** 2023-12-03

**Authors:** Alireza Zafarani, Mohammad Hossein Razizadeh, Atousa Haghi

**Affiliations:** a Department of Hematology & Blood Banking, School of Allied Medicine, Iran University of Medical Sciences, Tehran, Iran; b Department of Virology, School of Medicine, Iran University of Medical Sciences, Tehran, Iran; c Antimicrobial Resistance Research Center, Institute of Immunology and Infectious Diseases, Iran University of Medical Sciences, Tehran, Iran; d Young Researchers & Elite Club, Tehran Medical Sciences, Islamic Azad University, Tehran, Iran

**Keywords:** Influenza, Neutrophil, Neutrophil extracellular traps

## Abstract

Despite recent progress in developing novel therapeutic approaches and vaccines, influenza is still considered a global health threat, with about half a million mortality worldwide. This disease is caused by Influenza viruses, which are known for their rapid evolution due to different genetical mechanisms that help them develop new strains with the ability to evade therapies and immunization. Neutrophils are one of the first immune effectors that act against pathogens. They use multiple mechanisms, including phagocytosis, releasing the reactive oxygen species, degranulation, and the production of neutrophil extracellular traps. Neutrophil extracellular traps are used to ensnare pathogens; however, their dysregulation is attributed to inflammatory and infectious diseases. Here, we discuss the effects of these extracellular traps in the clinical course of influenza infection and their ability to be a potential target in treating influenza infection.

## 1 Introduction

Influenza is a contagious respiratory disease caused by the influenza virus. This disease is one of the most important global health issues and causes significant mortality worldwide, with more than half a million deaths annually [1]. Seasonal influenza epidemics occur during winter or other seasons of the year, based on geographical conditions. While the severity of influenza infection can vary widely, it is known to cause various symptoms such as fever, chills, dry cough, and sore throat. It can lead to pneumonia, acute respiratory distress syndrome (ARDS), and even death in high-risk individuals [2], including children under five years of age, the elderly, and patients with chronic comorbidities and weakened immune systems [3]. Thanks to high antigenic variation, influenza viruses change rapidly. Antigenic shift and drift are two mechanisms influenza viruses use to mutate and develop novel strains resistant to pre-existing immunity and medications [2,4].

Neutrophils are one of the most abundant effector cells of the innate immune system. Inflammatory responses caused by infections involve a quick migration of neutrophils from the bloodstream to the site of inflammation. Once there, neutrophils carry out various actions to eliminate microorganisms and resolve the infection. These actions include chemotaxis (movement towards chemical signals), phagocytosis (engulfing and destroying pathogens), the release of reactive oxygen species (ROS) and granular proteins, as well as the production and release of cytokines (immune signaling molecules). In addition to these established mechanisms, several studies have highlighted the significance of neutrophil extracellular traps (NETs) in this process [5]. NETs are an innate immune response involved in the pathogenesis of many infectious and non-infectious diseases. NETs are reticular structures composed of DNA, histones, and antimicrobial proteins such as neutrophil elastase and myeloperoxidase. Neutrophils release them in response to various stimuli, including bacterial and viral infections [6,7].

While NETs are important for host defense, their dysregulation has been implicated in various inflammatory diseases, including sepsis, rheumatoid arthritis, lupus, and cancer [8,9]. In some cases, NETs can exacerbate inflammation and tissue damage by promoting the release of anti-inflammatory cytokines and damaging host cells. NETs also play a role in blood clot formation, which can lead to thrombosis and other cardiovascular events [10,11].

Recent studies have shown that NETs may play a critical role in the pathogenesis of influenza infection. In this article, we review the effects of NETs in the pathogenesis of influenza and the potential effects of inhibiting NETs to reduce influenza symptoms.

## 2 Influenza viruses

Influenza viruses are members of the *Orthomyxoviridae* family. Four types of influenza viruses have been recognized, including A, B, C, and D. Influenza A and B viruses are responsible for most seasonal flu infections in humans. In contrast, influenza C viruses typically cause mild respiratory illness [12], and influenza D viruses primarily affect swine, caprine, and cattle and are not known to cause illness in humans [13].

Influenza viruses have a single-stranded RNA genome comprised of 8 segments with negative polarity. Along with the viral polymerase and nucleoproteins (NPs), this genome forms the ribonucleoprotein complexes (vRNPs). Influenza viruses have two major surface proteins, hemagglutinin (HA) and neuraminidase (NA), which are used to classify influenza viruses [14]. The immune response against HA and NA leads to the development of immunity [15]. Thus far, 18 different subtypes of HA and 11 different subtypes of NA have been detected, and influenza A viruses are named based on their HA and NA subtypes (e.g., H1N1, H3N2, etc.) [16].

Influenza viruses are highly infectious and can be transmitted within the population through respiratory droplets that are spread by talking, coughing, or sneezing [17]. The incubation period for influenza is typically 1–4 days [18], and symptoms can include fever, cough, sore throat, runny or stuffy nose, body aches, headache, chills, and fatigue. In some cases, influenza can also cause gastrointestinal symptoms such as nausea and diarrhea [2].

Influenza viruses are known to undergo rapid genetic changes, explaining new strains' emergence. This process is known as antigenic drift and occurs when mutations in the HA and NA genes accumulate over time. These changes may result in a different virus that can resist the immune system produced by previous vaccinations. In addition to antigenic drift, influenza A viruses can also undergo antigenic shift. This much more drastic genetic change can occur when two different influenza viruses exchange genetic materials in the same infected host. This process may lead to the emergence of an entirely new strain of virus that may cause a new pandemic [4].

Prevention of influenza involves vaccination, which is recommended for all individuals six months and older. The influenza vaccines are designed to protect against the influenza viruses that are expected to be most prevalent during the upcoming flu season. In addition to vaccination, other preventive measures include hand hygiene, avoiding close contact with infected individuals, and staying home if you are sick [18].

## 3 Neutrophil extracellular traps

NETs comprise chromatin fibers, histones, and granule proteins released by activated neutrophils in a " NETosis " process. Various physiological stimuli such as pathogens, antibodies, immune complexes, cytokines, and microcrystals can induce NETosis. When neutrophils undergo NETosis, they actively expel their chromatin and proteins to form a sticky NET, which can trap and kill pathogens [10].

So far, many types of NETs have been described, each with its own biochemical and functional characteristics. Suicidal NETs are a host defense mechanism in which neutrophils undergo a unique form of cell death, distinct from necrosis and apoptosis. When stimulated with certain chemicals or physiological factors such as phorbol myristate acetate (PMA) or IL-8, neutrophils undergo a stepwise process of chromatin decondensation, nuclear swelling, nucleoplasm spilling, and membrane perforation. Suicidal NETosis is an NADPH oxidase-dependent process that requires chromatin decondensation, followed by nuclear envelope disintegration and mixing of nucleic acids and granule proteins within a large vacuole. After intracellular assembly, suicidal NETs are released via plasma membrane perforation and cell lysis. The released NETs can bind and kill gram-negative and gram-positive bacteria, making them a powerful host defense mechanism [19].

Vital NETs are a form of NETs release that allows for the coexistence of NETosis and conventional host defense, unlike suicidal NETosis. Vital NETosis is triggered by microbial-specific molecular patterns recognized by host pattern recognition receptors and is mediated by Toll-like receptor 4 (TLR4) on platelets that facilitate activation of PMNs, making it a rapid process. The functional capacity of the PMNs during NET release is maintained in vital NETosis, and the cells become anuclear cytoplasts capable of chasing and imprisoning live bacteria. Additionally, granulocytes may have both a lytic and nonlytic pathway of NETosis, making vital NETosis an essential pathway for host defense against microbial pathogens [20].

### 3.1 Functions of NETs

The functions of NETs are multifaceted and complex and are still being investigated by researchers. One of the main functions of NETs is to trap and kill pathogens such as bacteria and fungi. Antimicrobial DNA and proteins in NETs can bind to these pathogens and inactivate them, prevent their spread, and prevent the destruction of host tissues. This process is known as "NETosis" and is considered a critical part of the extracellular immune response to microbial infections. Being a conserved process among various species, NETosis is considered as an evolutionary advantage of innate immunity [21].

NETs and complement systems act hand-to-hand to defend the host against infectious diseases. NETs are involved in the modulation of the complement system response in infectious diseases since they are one of the danger-associated molecular patterns (DAMPs) [22]. The complement-neutrophil interaction is of uttermost importance in the pathogenesis of anti-neutrophil cytoplasmic antibody (ANCA)-associated vasculitis. ANCAs can trigger neutrophils to release NETs [23]. Additionally, complement opsonization enhances NET generation [24]. It has been shown that the terminal complement activation complex C5b-9 can stimulate the release of NETs [25].

The function of NETs in bacterial infections remains a subject of ongoing investigation. Initially, it was thought that NETs aided in eliminating bacteria by ensnaring and potentially eradicating these pathogens. However, emerging research suggests that NETs might primarily impact bacterial growth, ultimately contributing to their elimination. The DNA within NETs exerts antimicrobial effects through cation chelation and disrupting cell membranes. Notably, NETs can ensnare bacteria without necessarily inducing their demise while influencing the complement proteins' ability to neutralize them [26,27].

Although bacterial infections are established triggers for NET production, recent findings indicate that viruses also can stimulate NET release. Recognition receptors on neutrophils, including those within endosomes, play an important role in initiating NET formation after facing pathogens. Viruses, similar to bacteria, may adopt strategies to counteract NET release mechanisms during infection. Interestingly, the pathways viruses induce NET release can differ depending on the specific pathogen, potentially leading to distinct outcomes. Furthermore, the impact of NET release in viral infections can be intricate, occasionally either supporting or deterring virus-induced damage. This evolving comprehension underscores the complex and multifaceted role of NETosis in bacterial and viral infections [7,26,27]. Intriguingly, the compositions of NETs induced by viral infections differ from those induced by bacterial infections. Moreover, it has been observed that influenza-induced NETosis does not protect against secondary bacterial infections [7].

### 3.2 Biogenesis of NETs

The formation of NETs, a process called NETosis, involves a series of steps that lead to the release of nuclear DNA and associated proteins from the neutrophil into the extracellular space. Suicidal NETosis is a cell death program apart from apoptosis and necrosis (19). Activated neutrophils release NETs in response to various stimuli, such as pathogens, cytokines, and immune complexes. The first step in NETosis is the activation of neutrophils. Protein kinase C (PKC) increases ROS formation upon activation through the NADPH oxidase complex. Neutrophil elastase and myeloperoxidase are translocated from the cytoplasm to the nucleus, where they play a crucial role in chromatin decondensation [28].

The process of chromatin decondensation is necessary for the formation of NETs. During this process, peptidyl arginine deiminase 4 (PAD4) is activated, which converts arginine residues within histones to citrulline. This citrullination event disrupts the ionic interactions between histones and DNA, making chromatin more accessible to the neutrophil's cytoplasmic granules [29].

After chromatin decondensation, the neutrophils undergo cytoplasmic granule mobilization. This mobilization process transports various antimicrobial peptides and enzymes from the granules to the cell's periphery. The granules contain proteins such as neutrophil elastase, myeloperoxidase, and lactoferrin, which are involved in host defense [30].

The final step in NETosis is the membrane rupture process, which releases NETs into the extracellular space. This process is regulated by activating PKC, which phosphorylates numerous neutrophil cytoskeletal proteins. The NETs cast into the extracellular space can entrap and kill invading pathogens, playing a crucial role in host defense [30].

## 4 Neutrophil extracellular traps and influenza comorbidities

NETs have emerged as a double-edged sword in various infections, including influenza. It is a defensive mechanism that protects the body by secreting cytokines and antimicrobial peptides as well as recruiting other immune cells to the site of infection. NETs' beneficial role in viral infection has been observed; for example, in poxvirus infection, the introduction of virus analogs through systemic administration or infection with poxviruses triggers the recruitment of neutrophils to the liver's small blood vessels (microvasculature). These neutrophils release NETs, which act as a defensive mechanism by safeguarding host cells from viral infection [31]. In the case of the influenza infection, histone H3 and H4, rich in arginine, have been found effective against the influenza virus [32]. On the other hand, NETosis plays an important role in lung tissue damage in the course of influenza virus infection [33].

Recent studies have shown that the generation of NETs in response to influenza infection significantly contributes to the severity of the disease.

When an influenza infection imposes the formation of NETs, the DNA and protein components of the NETs can cause significant damage to the lung tissue. This damage can result in the promotion of inflammation and a decrease in gas exchange. The formation of NETs can also act as a scaffold for the concentration of immune cells. The accumulation of these immune cells in the lungs can cause further inflammation and damage lung tissue [34].

Additionally, when NETs trap and kill influenza viruses, they can release viral components, such as RNA and DNA, into the extracellular space. These viral components can increase inflammation and contribute to lung tissue damage. The viral components in the extracellular space can also trigger the immune system, causing an overreacted immune response that can damage the lung tissue [34].

Accumulating data have suggested that influenza infection can result in a hyperinflammatory response and cytokine storm, which can cause damage to tissues and organs, resulting in multiple organ failures [35]. NETs have been found to play a vital role in the inflammatory response by awaking the complement system and increasing the generation of pro-inflammatory cytokines such as interleukin-1, interleukin-6, and tumor necrosis factor (TNF). In COVID-19, research has demonstrated that the SARS-CoV-2 virus can directly stimulate neutrophils, releasing NETs. This activation can occur through various means, including the virus binding to toll-like receptors on neutrophils or causing damage to endothelial cells, which exposes certain components of the extracellular matrix that trigger NET release. The excessive production of NETs in individuals with COVID-19 contributes to developing a cytokine storm through multiple mechanisms. Mainly, NETs can directly activate and induce the production of pro-inflammatory cytokines such as IL-1β, IL-6, and TNF-α. These cytokines further recruit and activate immune cells, amplifying the overall inflammatory response [36].

ARDS is an essential and life-threatening consequence of influenza infection defined by acute lung injury, hypoxemia, and respiratory failure [37,38]. By promoting the production of pulmonary thrombi and activating the coagulation cascade, NETs have been linked to the development of ARDS [37]. It has been demonstrated that individuals admitted with greater levels of plasma NETs presented with more severe influenza infection. These patients' neutrophils typically discharge more DNA complex [39]. The levels of NETs were higher in the plasma and bronchoalveolar fluid of patients who had ARDS due to transfusion or pneumonia than those who did not [40]. It has been demonstrated that COVID-19-induced NETs are associated with immunothrombosis and more severe ARDS; they also showed that neonatal NET-inhibitory factor (nNIF) is a helpful treatment in COVID-19-induced NETs which maybe also helpful in influenza induced ARDS [41]. Massive recruitment of neutrophils to the lung caused severe ARDS in a mouse model of coinfected influenza and bacterial infection. The inhibition of NET formation by DNase I reduced lung inflammation and improved survival [42]. In another mouse study, macrophage-depleted mice displayed more severe ARDS-related symptoms than neutrophil-depleted mice, demonstrating the significance of NET formation in ARDS severity [43]. The study also indicated that NETs caused by influenza do not protect against secondary bacterial infections [44]. It is essential to note that ARDS is acute and unpredictable; providing a solid guideline that contains the timing, type, and dose of treatments is urgent for each medical center based on its infrastructure. Treatment with the pyrimidine synthesis inhibitor A77-1726 on days 1 and 5 after infection reduced viral replication on Day 6 and enhanced alveolar fluid clearance, peripheral oxygenation, and cardiac function [45]. However, in the case of NETosis inhibitors, there is a paucity of studies on the timing of these drugs in influenza-related ARDS.

Sepsis is another severe medical condition related to NETs that happen when the immune system overreacts to an infection, leading to inflammation throughout the body, which can damage organs and tissues and cause organ failure [46]. Studies have shown that NETs play a significant role in developing sepsis. NETs can trigger platelet activation and adhesion, leading to blood clots or thrombi formation. These clots can cause ischemia or restrict blood supply to tissues, leading to organ dysfunction and failure [47]. In a mouse model of sepsis, researchers found that the hampering of the NET formation using DNase I reduced platelet activity and improved survival. This suggests that NETs directly impact platelet activation and thrombus formation in sepsis. By inhibiting the formation of NETs, platelet activity can be reduced, leading to a decrease in the development of thrombi and improving survival rates [48].

**Fig. 1:**
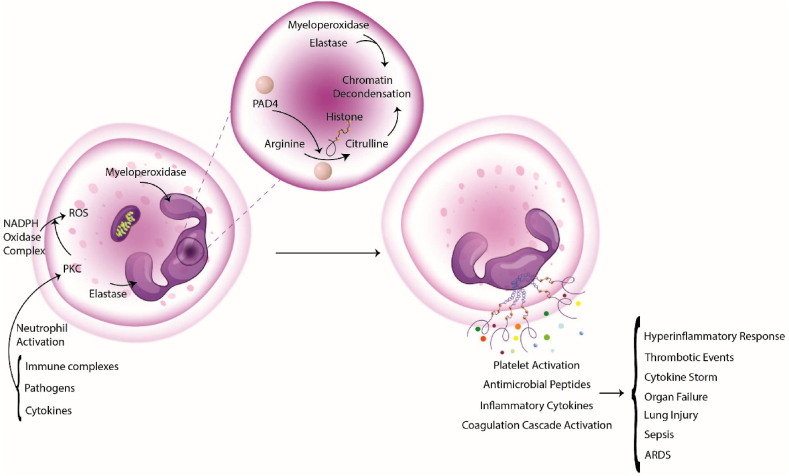
The protein kinase C (PKC) pathway is activated upon activation, leading to increased reactive oxygen species (ROS) production through the NADPH oxidase complex. This, in turn, leads to the translocation of neutrophil elastase and myeloperoxidase from the cytoplasm to the nucleus, where they play a crucial role in chromatin decondensation. This process involves the activation of PAD4, which converts arginine residues within histones to citrulline. This citrullination event disrupts the ionic interactions between histones and DNA, making chromatin more accessible to the neutrophil's cytoplasmic granules. Following chromatin decondensation, the neutrophils undergo cytoplasmic granule mobilization. The final step in NETosis is the membrane rupture process, which leads to the release of NETs into the extracellular space. NETs lead to comorbidities like a hyperinflammatory response, thrombotic events, cytokine storm, organ failure, lung injury, sepsis, and ARDS.

Influenza infection promotes the risk of thrombotic events, particularly in high-risk individuals such as the elderly and those with underlying cardiovascular disease [49]. NETs have been shown to play a role in developing thrombotic events by enhancing platelet activation, microthrombi formation, and coagulation cascade activation [50]. Additionally, influenza virus infection can increase the growth of tissue factor (TF), a protein that promotes blood clot formation. The release of NETs in response to influenza virus infection can further enhance the procoagulant state by increasing platelet activation and fibrin production, thus contributing to the development of thrombosis [41]. Studies have found that NETs contribute to developing thrombosis during influenza infection. In a study on mice with influenza pneumonitis, researchers investigated the role of neutrophils and NETs in developing acute lung injury. The study showed that the influenza virus triggered the release of NETs, which caused platelet activation and increased fibrin production. The study also found that higher levels of neutrophil infiltration and the formation of NETs were associated with more severe lung injury and increased mortality rates in infected mice [43]. The researchers realized that the lungs of infected mice were filled with many NETs, promoting thrombin formation through platelet-dependent and platelet-independent mechanisms. The research showed that hampering NET formation using DNase I decreased thrombin production and the risk of thrombosis in infected animals [51]. A summary of NETosis in influenza comorbidities is shown in Fig. 1.

In the case of COVID-19, another respiratory viral infection, researchers evaluated the interaction between complement and the platelet/NETs/thrombin axis in COVID-19 patients using patients' specimens, cell-based inhibition tests, and NET/human aortic endothelial cell (HAEC) co-cultures have been examined. They found increased levels of NETs, TF activity, and sC5b-9 in the patient's plasma. Neutrophils from patients showed high TF expression and released NETs with active TF. Treatment of control neutrophils with COVID-19 platelet-rich plasma produced TF-bearing NETs that induced thrombotic activity in HAECs. However, inhibiting thrombin or NETosis or blocking C5aR1 reduced platelet-mediated NET-driven thrombogenicity. COVID-19 serum activated complement *in vitro*, consistent with high complement activity in clinical samples. Inhibiting complement C3 with compstatin Cp40 disrupted TF expression in neutrophils, suggesting this drug as a potential option for NET-induced thrombosis [52]. Besides thrombosis, inhibiting the complement system can abate the NETosis since complement components can trigger it. It has been observed that Eculizumab, a humanized monoclonal antibody impedes the cleavage of C5 to C5a and C5b plunged the NETosis in patients with paroxysmal nocturnal hemoglobinuria (PNH) [53].

## 5 Targeting neutrophil extracellular traps to cure influenza infection

Targeting NETs to treat influenza infection is an emerging therapeutic strategy with great potential. Several approaches have been proposed to achieve this, including preventing the NET formation and using drugs that target the proinflammatory cytokines released due to the NET release.

The inhibition of NET formation has been investigated as a potential therapy for influenza infection. The endonuclease enzyme DNase-1 may hold promise as a treatment for influenza because it specifically targets the DNA in extracellular NETs. Its function in dismantling the NETs' DNA framework is essential for removing these structures by phagocytic cells like macrophages. By effectively preventing the emergence of NETs, this mechanism also lessens the severity of several NET-related comorbidities, including sepsis, thrombosis, and autoimmune disease. Exogenous DNase-1 administration could also be a practical therapeutic approach for relieving these illnesses [54,55].

In addition to DNase I, different pharmacological compounds have been investigated as potential inhibitors of NETs, which can be considered for treating influenza-related comorbidities. Cl-amidine is a small molecule inhibitor of PAD4. Studies have shown that the activity of PAD4 can be effectively inhibited by Cl-amidine, thus preventing the NETs. Cl-amidine has been shown to positively affect several disease models, such as lupus nephritis, multiple sclerosis, and sepsis, by inhibiting NETs [[56], [57], [58]]. Antioxidative N-acetylcysteine (NAC) has been shown to have several positive health effects. Inhibiting the development of NETs is one of the hypothesized mechanisms by which NAC may lessen the severity of influenza infection [54]. NAC has been shown to have reduced NETs formation in healthy neutrophils and myeloproliferative neoplasm (MPN) neutrophils [59]. A study investigated the effects of long-term NAC treatment on the immune response and symptoms of individuals with influenza-like illnesses. The study involved 262 randomly assigned individuals receiving 600 mg of NAC or a placebo twice daily for six months. The findings demonstrated that the group receiving NAC significantly decreased the severity and frequency of influenza-like symptoms compared to the placebo group. The NAC group also exhibited enhanced cell-mediated immunity as indicated by elevated T-lymphocyte and natural killer cell counts. According to the authors, long-term NAC therapy may be a secure and efficient method for boosting immune system health and lessening the severity of influenza-like illness [60]. A recent study found that histones have anti-influenza activity against seasonal H3N2 and H1N1 strains but not pandemic H1N1. Histones rich in arginine, such as H3 and H4, were found to have greater neutralizing and viral aggregating activity than those rich in lysine, such as H2A and H2B. Among all core histones, histone H4 was the most potent in neutralizing influenza, and it inhibited viral replication in epithelial cells. The antiviral activity of histone H4 was mainly due to its direct effects on viral particles. The study showed that H4 binds to influenza and induces aggregation. However, H4 was not effective against pandemic H1N1 [61]. The scientists discovered that histones could promote inflammation and lung damage in mice infected with the influenza virus. They also found that treating mice with an antibody that neutralizes histones reduced lung damage and improved survival rates. The study suggests that targeting extracellular histones could be a potential strategy for treating severe influenza infections [62].

When the body responds to an influenza virus infection, it releases NETs, activating the innate immune system and producing proinflammatory cytokines and antimicrobial peptides like IL-6, IL-8, LL-37, and TNF-α. These cytokines can worsen the condition. To treat influenza, one possible method is to target these cytokines and reduce their production [38,[63], [64], [65]]. For example, researchers investigated the optimal timing of treatment with a CXCR2 antagonist, SCH527123, and its effectiveness in combination with the antiviral agent, oseltamivir, in mice and piglet influenza-pneumonia models. The combination treatment improved survival rates in mice and reduced lung damage in piglets by attenuating neutrophil influx and NETosis. The researchers found neutrophils from influenza-infected mice were more susceptible to NETotic death when stimulated with a CXCR2 ligand, IL-8. CXCR2 stimulation also induced nuclear translocation of neutrophil elastase and enhanced histone citrullination, which triggers chromatin decondensation during NET formation. These findings provide important insights into the optimal timing of CXCR2 antagonist treatment to attenuate neutrophil-mediated lung pathology during influenza infections, suggesting that pharmacologic treatment with a CXCR2 antagonist and an antiviral agent could significantly improve outcomes in severe and mild influenza infections [66]. New research indicates that LL-37, a human cathelicidin, can effectively combat influenza by displaying both *in vitro* and in vivo antiviral activity. While LL-37 did not affect neutrophil uptake of influenza, it significantly increased neutrophil H2O2 responses to the virus. *In vitro*, influenza induced the production of NETs, and preincubation of the virus with LL-37 increased this response [67].

Moreover, additional possible targets for influencing the development and function of NETs are presently being investigated. Many studies have identified the receptor for advanced glycation end products (RAGE) as a critical regulator of NET production [68]. When advanced glycation end products (AGEs) accumulate in the body, they can bind to RAGE on neutrophils, which triggers the release of NETs [68]. It has been shown that inhibiting RAGE reduces NET release *in vitro* and in vivo [68]. Neutrophil elastase, which is involved in the degradation of extracellular matrix proteins and has been linked to the development of pulmonary fibrosis and other tissue damage associated with influenza infection, is another possible target [69].

A study stimulated human neutrophils with PMA or *Staphylococcus aureus* and measured NETs formation, bactericidal effect, ROS production, and NF-κB activation. They found that Dexamethasone (DXM) inhibited *S. aureus*-induced NETosis and bacterial killing, but not PMA-induced NETosis, and that TLR2 and TLR4 modified *S. aureus*-induced NETs formation. DXM did not affect oxidant production or NF-κB activation, suggesting a TLR-dependent mechanism. Agonists of TLR2 and TLR4 rescued DXM-inhibited NETosis, while antagonists did not further inhibit the reduction induced by DXM, indicating that DXM may inhibit NETosis by regulating TLR2 and TLR4 [70].

Calcineurin is a calcium-dependent serine/threonine protein phosphatase that plays a considerable role in neutrophil activity, such as NET formation, since many stages of NETosis depend on calcium mobilization [71]. A study found that extracellular and intracellular calcium pools are required to induce NETosis efficiently. Drugs targeting the calcineurin pathway, such as cyclosporine A, can reduce NETosis. Ascomycin and cyclosporin A also indicated promising effects in reducing IL-8-induced NETosis [72]. These findings suggest that it may be possible to suppress or modulate NETosis with pharmacological treatments [72].

In Table 1 we list some potential ROS scavengers as a potential treatment. It has been shown that Methotrexate can inhibit ROS formation; as a result, it may be a treatment option for the inhibition of NETs indirectly [73]. The role of antioxidants in NETs inhibition has been investigated. A study found that Trolox and Tempol effectively inhibited ROS-dependent NET release, while Tiron had no significant inhibitory effect [74]. Octyl gallate (OG) has been recently introduced as a potential inhibitor of ROS and NETs, which can be nominated for inhibition of NETs in different diseases, including influenza [75]. Diphenyleneiodonium chloride (DPI) is another possible compound inhibiting NETs formation [76].

**Table 1:** List of therapeutic approaches with the potential to cure Influenza-induced NETosis.

Medication type	Target	Activity	Example
TLR inhibitors	TLRs	Impedes TLR activity leaving ROS production unaffected	Dexamethasone [70]
Calcineurin Inhibitors	Calcineurin pathway	Reducing neutrophil activity by disrupting calcium pathway	Cyclosporine A [72]
ROS scavengers	ROS	Reducing NETosis by decreasing mitochondrial ROS formation	N-acetylcysteine [59] Methotrexate [73] Trolox and Tempol [74] Octyl gallate [75] Epigallocatechin gallate [77] Diphenyleneiodonium chloride [76]
PAD inhibitors	PAD	Acting against NETosis by inhibiting PADs.	YW3-56 [78]
DNase	DNA	Degrading DNA and inhibiting neutrophil infiltration	DNase-1 [83,84]
IL-6 and 8 receptor antagonists	IL-6 and IL-8	Obstructing IL-6 and IL-8 pathways to induce NETosis	Tocilizumab [64] Reparixin [65]
Complement inhibitors	The complement system components	Inhibiting complement-induced NETosis	Eculizumab [53]
Vitamins	Neutrophils	Increases clearance by macrophages, and inhibits necrosis Preventing endothelial damage	Vitamin C [79] Vitamin D [80]

Scientists induced severe acute pancreatitis (SAP) in mice and inhibited NE activity using GW311616. They found that inhibiting NE activity reduced NET formation, tissue damage, and inflammatory responses in SAP. They also identified epigallocatechin-3-gallate (EGCG) as a potential therapeutic compound using network pharmacology and molecular docking. The researchers demonstrated that EGCG could inhibit NE activity and reduce NET formation *in vitro* and in vivo, thereby reducing tissue damage and inflammation in SAP [77].

Researchers investigated the molecular mechanisms involved in PAD and NET formation during endotoxic stress in mice. They administered lipopolysaccharide to induce endotoxic shock and treated some mice with a PAD inhibitor called YW3-56. The results showed that YW3-56 significantly increased the survival time of mice with endotoxic shock. They also found that YW3-56 inhibited NET formation, reduced the levels of cytokines (IL-6, TNFα, and IL-1β), and decreased lung tissue injury [78].

The role of vitamins like C and D has been investigated in NETs inhibition. For example, The active form of Vitamin D, known as 1,25(OH)2D3, may potentially reduce damage to the inner lining of blood vessels by reducing NETs. This finding suggests that Vitamin D could be used as an additional treatment for patients with SLE who have low levels of Vitamin D to prevent damage to their blood vessels [79,80]. In Table 1, a list of possible drugs that can be used to alleviate NETosis adverse effects in influenza disease is presented.

nNiF is an endogenous NETosis inhibitor that targets key events in NET formation, including PAD4 activation, chromatin decondensation, and nuclear envelope breakdown in neonates and adults. nNIF is a 38-amino acid peptide derived from the C-terminal region of the human protein, leucine-rich alpha-2-glycoprotein-1 (LRG1). It showed favorable outcomes in treating polymicrobial sepsis's cecal ligation and puncture model [81]. Additionally, other molecules named nNIF-related peptides (NRPs) have been identified with the ability to inhibit NETosis triggered by pathogens, microbial toxins, and pharmacologic agonists *in vitro* and in mice [82].

In addition, it is essential to remember that the release of NETs is a vital host defensive mechanism against viruses and that preventing their creation may interfere with the body's natural defenses. To avoid interfering with their positive roles in host defense while preventing their pathological repercussions in influenza infection, a balanced strategy is essential when targeting NETs.

## 6 Conclusion

One of the strategies used by the innate immune system to defend the body from pathogens is the sacrifice of neutrophils to release NETs. However, NETs can be detrimental and, instead, contribute to the pathogenesis. The emerging evidence on the role of NETs in the pathogenesis of influenza and its comorbidities highlights the need for new therapeutic strategies that target the underlying inflammatory and thrombotic mechanisms. While the inhibition of NET formation and function represents a promising approach, further research is needed to evaluate the safety and efficacy of this strategy in human clinical trials. Ultimately, a multi-faceted approach that includes vaccination, antiviral therapy, and supportive care is likely the most effective way to reduce the morbidity and mortality associated with influenza and its comorbidities.

## Data availability statement

Data used in this article are included in references.

## CRediT authorship contribution statement

**Alireza Zafarani:** Writing – review & editing, Writing – original draft. **Mohammad Hossein Razizadeh:** Writing – review & editing, Writing – original draft, Conceptualization. **Atousa Haghi:** Writing – review & editing, Writing – original draft, Visualization.

## Declaration of Generative AI and AI-assisted technologies in the writing process

The authors utilized ChatGPT version 3.5 (OpenAI LP, CA) to enhance the language readability of the manuscript using the prompt: Tell the grammtical problems of the text.

## Declaration of competing interest

The authors declare that they have no known competing financial interests or personal relationships that could have appeared to influence the work reported in this paper.
